# Activity demands and instability are the most important factors for recommending to treat ACL injuries with reconstruction

**DOI:** 10.1007/s00167-018-4846-1

**Published:** 2018-02-06

**Authors:** Hanna Tigerstrand Grevnerts, Anne Fältström, Sofi Sonesson, Håkan Gauffin, Siw Carlfjord, Joanna Kvist

**Affiliations:** 1 Department of Activity and Health, Linköping University, 58183 Linköping, Sweden; 2 Division of Physiotherapy, Department of Medical and Health Sciences, Linköping University, 581 83 Linköping, Sweden; 3 Region Jönköping County, Rehabilitation Centre, Ryhov County Hospital, Jönköping, Sweden; 4 Division of Social Sciences, Department of Medical and Health Sciences, Linköping University, 581 83 Linköping, Sweden; 5 Orthopaedic Department Linköping University Hospital, Department of Clinical and Experimental Medicine, Linköping University, 581 83 Linköping, Sweden

**Keywords:** Knee, Anterior cruciate ligament injury, Treatment decision, ACL reconstruction

## Abstract

**Purpose:**

The purpose of the study was to (1) study and compare the factors that Swedish orthopaedic surgeons and physical therapists consider important for recommending ACL reconstruction and, (2) to assess how orthopaedic surgeons and physical therapists consider their own and each others, as well as patients’, roles are in the treatment decision.

**Methods:**

A web-based survey assessing the relevance of 21 predetermined factors, in the choice to recommend ACL reconstruction, was sent to orthopaedic surgeons and physical therapists. Respondents were also asked to rate the importance of the assessment made by themselves, the other clinician (physical therapists rated the importance of surgeons, surgeons rated the importance of physical therapists), and the patients’ preferences.

**Result:**

Orthopaedic surgeons agreed of eight, and physical therapists of seven factors as important in the choice to recommend ACL reconstruction. The factors both groups reported as important were; “patient’s wishes to return to contact/pivoting sports”, “instability in physical activity”, “instability in activities of daily living despite adequate rehabilitation”, “physically demanding occupation”, and “young age”. Both professions rated their own and each others assessments as well as patient’s wishes as important for the decision to recommend ACL reconstruction.

**Conclusion:**

Orthopaedic surgeons and physical therapists agree about factors that are important for their decision to recommend ACL reconstruction, showing that both professions share a common ground in perceptions of factors that are important in recommending ACL reconstruction.

**Level of evidence:**

Diagnostic study: Level III.

**Electronic supplementary material:**

The online version of this article (10.1007/s00167-018-4846-1) contains supplementary material, which is available to authorized users.

## Introduction

An anterior cruciate ligament (ACL) injury is often treated initially with physical therapy, and in many cases followed by an ACL reconstruction (ACL-R) plus post-operative rehabilitation [22]. Today, there is no clear evidence of which treatment, surgery followed by rehabilitation or rehabilitation alone, is superior, or on which basis either treatment should be chosen. Noyes proposed in the 1980s that one-third of ACL-injured patients would function well without surgical treatment [22, 23]. In a recent randomised clinical trial, there was equivalent self-reported outcome at 2 and 5 years after injury among patients treated with ACL-R or rehabilitation only [9, 10]. A treatment decision algorithm, based on clinical performance tests and patient-reported outcome questionnaires, aiming to identify appropriate patients for surgical or nonsurgical treatment has been developed at the University of Delaware [7, 13, 14]. Still, when using the screening algorithm, 70% of the patients who chose not to undergo ACL-R despite being identified as appropriate candidates for surgical treatment, showed good patient-reported outcome and had returned to their previous activity level at 1 year follow-up [21]. This implies that identifying the right treatment for the right patient is complex and challenging.

There are several guidelines published from different countries, where knee joint instability, sporting demands or insufficient/failed rehabilitation, are important factors impacting on the decision to recommend ACL-R [2, 18, 20, 25].

The importance of providing rehabilitation treatment for this group of patients (with or without surgery), underscores the role of the physical therapist as an important caregiver [4, 16]. The physical therapist’s role in the treatment decision has not yet been explored. There is also a need to study the factors that physical therapists consider are important to the recommendation for ACL-R, and how orthopaedic surgeons and physical therapists rate the importance of each other’s assessment in the choice of treatment. Physical therapists work closely with patients through rehabilitation, and may influence patients’ views regarding preferred treatment. So, it is important to understand physical therapists’ views regarding how treatment decisions are made. This information might also enhance collaboration between orthopaedic surgeons and physical therapists. For example, physical therapists often have detailed knowledge of the patient’s objective knee function; knowledge that might be of value in the treatment decision making process.

The aims of this descriptive survey study were: (1) to study and compare the factors Swedish orthopaedic surgeons and physical therapists consider important for recommending ACL-R, and (2) to assess how important orthopaedic surgeons and physical therapists consider their own and each others’, as well as patients’, roles are in the treatment decision.

## Materials and methods

In this descriptive study, an online survey (Online Appendix 1, Online Appendix 2) was sent to orthopaedic surgeons and physical therapists who were active in treating patients with ACL injury.

### Survey

The first draft of the survey was constructed by a group of researchers with extensive research experience in ACL injury. The predetermined factors were based on research by Swirtun et al. [27], that revealed factors that patients believed to be important for the choice to undergo ACLR. These factors were influential to the factors that has been used in other studies (unpublished material) and reviewed by researchers, before use in the present survey. To obtain face validity, the survey was sent to four orthopaedic surgeons (active in spine, shoulder and achilles tendon surgery, one with previous experience in ACL-R), and five physical therapists (two with extensive experience in treating patients with ACL injuries and three with experience in general musculoskeletal disorders). The orthopaedic surgeons and physical therapists reviewed the questions and wording for content and clarity. Their comments lead to some minor changes and clarifications of the survey.

The final survey that was sent to orthopaedic surgeons and physical therapists contained sociodemographic questions, questions about experience in treating patients with ACL injury, knowledge of ACL treatment guidelines, and 21 predetermined factors (Online Appendix 1, Online Appendix 2) that should be graded regarding importance to recommend ACL-R. The predetermined factors were on a 0–3 scale, were a higher grade corresponds to higher likeliness to recommend ACL-R. There was an open-ended question about which factor or combination of factors were considered the most important factors for the clinician to recommend ACL-R. Questions about patient rehabilitation and how physical therapists present their assessment findings to the orthopaedic surgeon, were included, as well as questions about how important the physical therapists and orthopaedic surgeons consider their own and the other profession’s assessment, and the patient’s wishes.

### Study participants

Almost all (> 90%) orthopaedic surgeons who perform ACL-R in Sweden register their operative procedures in the Swedish national knee ligament register [28].. All surgeons registered as conducting ACL-R in the register, were contacted by email (236 email addresses). The number of surgeons actively performing ACL-R during 2013 and 2014, when the survey was conducted, was 160 and 152, respectively, implying that some registered email addresses might be to inactive surgeons or some surgeons might have had multiple registered email addresses. One email address was incorrect, resulting in 235 invitation email surveys being sent to orthopaedic surgeons.

Physical therapists that were potentially active in treating patients with ACL injury were identified by the Swedish Society for Physical Activity and Sports Medicine, Swedish Football Physiotherapists Association, orthopaedic clinics over Sweden and through professional contacts. After the survey was sent, several physical therapists contacted the research group and gave email addresses for colleagues they knew treated patients with ACL injury (snowball recruitment), resulting in two additional rounds of mailings. In total, 951 emails were sent. Of the 951 physical therapists’ email addresses, four were incorrect resulting in 947 invitation email surveys being sent to physical therapists. There were no checking to see if there were multiple addresses for a single respondent, and therefore, no certainty that there were 947 individual physical therapists who were approached to participate.

To improve generalizability, we invited orthopaedic surgeons and physical therapists who worked in the larger cities and smaller regions and counties in Sweden.

### Data collection

All surveys were sent in April 2014. Up to two reminders were sent to participants who had not responded. The surveys were distributed by a secure web-based survey system (esMaker): version 3.0^©^ Entergate AB, and were completed and returned anonymously.

This study had ethical approval from the Regional Ethical Review Board Dnr 2014 (71/31).

### Data analysis

For ratings of the factors influencing the choice to recommend ACL-R, we dichotomized the responses to “no surgery” for orthopaedic surgeon and “little probability that I would recommend ACL reconstruction” for physical therapists (when the rating was 0 or 1) or “surgery” for orthopaedic surgeons and “great probability that I would recommend ACL reconstruction” for physical therapists (when the rating was 2 or 3) (Online Appendix 1, Online Appendix 2). If a respondent answered “surgery”/“great probability that I would recommend ACL reconstruction”, we interpreted this as indicating that the factor was important for recommending ACL-R, and subsequently used the factor in the analysis of factors reaching clinical agreement.

Clinical agreement was defined as when 80% of respondents had chosen the same response option (after dichotomizing the answer options) [19].

We categorised answers to the open-ended question of factors that were important for choosing ACL-R into 1 of 6 categories (instability, patient focus, activity demands, other injuries, sociodemographic factors and objective measures). These categories were based on the experience from previous studies within the research group, and after reviewing the answers from this study. Categorization (and any further sub-categorization) was made by a physical therapist (HTG) with both clinical and research experience in treating ACL injuries. For each category, we examined whether it was described as a sole factor in the decision to recommend ACL-R, or as part of a cluster of factors that were considered together by the clinician as a basis for the decision to recommend ACL-R.

## Results

There were 130 orthopaedic surgeons who responded to the email invitation (Table 1). Among these, 98 (75%) accepted to participate, 7 declined (5%) due to unspecified reasons, and 25 (19%) declined due to not treating patients with ACL injury. Of the nonresponding orthopaedic surgeons there were 11 women and 94 men.

**Table 1:** Sociodemographic data and experience in treating patients with ACL injury among responding orthopaedic surgeons and physical therapists

	Orthopaedic surgeons Total: 98	Physical therapists Total: 391
	*n* (%)	*n* (%)
Gender (M/F)	88 (90)/10 (10)	186 (48)/205 (52)
Age (in years)		
< 30	0 (0)	57 (15)
31–45	38 (39)	121 (31)
46–55	31 (32)	144 (37)
> 56	29 (30)	68 (17)
Experience^a^ (in years)		
< 2	7 (7)	31 (8)
2–5	14 (14)	64 (17)
6–10	19 (19)	58 (15)
> 10	58 (59)	232 (60)
Patient volume during the last 6 months^b^		
0	5 (5)	18 (5)
1–4	5 (5)	119 (31)
5–15	37 (38)	175 (45)
> 15	51 (52)	76 (20)
Knowledge of guidelines^c^		
International	18 (18)	50 (13)
National	44 (45)	107 (27)
Local	63 (64)	237 (61)
No knowledge	16 (16)	111 (28)

*ACL* anterior cruciate ligament, *ACL-R* anterior cruciate ligament reconstruction

^a^Experience (in years) for conducting anterior cruciate ligament reconstruction (surgeons) or for treating patients with an ACL injury (physical therapists)

^b^The number of ACL-R performed (orthopaedic surgeons), or ACL-deficient patients treated (physical therapists) during the last 6 months

^c^The response options of whether the respondent had knowledge of guidelines guidelines/health care programmes outlining which patients should undergo an ACL-R was; “no”, “yes international guidelines” “yes national guidelines” and “yes local guidelines”. Multiple answers were allowed for the question

There were 595 physical therapists who responded to the email invitation (Table 1). Among these, 391 (66%) accepted to participate, 38 (6%) declined due to unspecified reasons, and 166 (28%) declined due to not treating patients with ACL injury. Of the 352 nonresponding physical therapists, 201 were women and 151 were men.

Regarding the factors influencing the treatment decision-making process, orthopaedic surgeons reached clinical agreement for 8 factors, and physical therapists reached clinical agreement for 7 factors (out of a total of 21 factors). (Table 2).

**Table 2:** Factors rated for their importance to recommend ACL-R

Factors		*N* ^a^	Orthopaedic surgeons Total (*n* = 98)			*N* ^a^	Physical therapists Total (*n* = 391)		
			Rate 2 or 3 *n* (%)	Rate 2 *n*	Rate 3 *n*		Rate 2 or 3 *n* (%)	Rate 2 *n*	Rate 3 *n*
Patient age									
< 25	**91**		**81 (89)**	**36**	**45**	**370**	**298 (80)**	**144**	**154**
25–40	90		68 (76)	50	18	356	234 (66)	175	59
> 40	90		24 (27)	19	5	354	79 (22)	59	20
Open physes	91		39 (43)	27	12	368	70 (19)	57	13
Sex									
Male	90		69 (77)	31	38	360	246 (68)	148	98
Female	90		70 (78)	30	40	360	245 (68)	144	101
Wish to return to sport									
Contact/pivoting									
Elite level	**91**		**90 (99)**	**10**	**80**	**377**	**366 (97)**	**40**	**326**
Recreational	**91**		**87 (96)**	**33**	**54**	**375**	**321 (86)**	**150**	**171**
Non-contact/non-pivoting									
Elite level	91		64 (70)	37	27	374	250 (67)	163	87
Recreational	91		39 (43)	25	14	371	75 (20)	59	16
Occupation	**92**		**88 (96)**	**18**	**70**	**373**	**324 (87)**	**150**	**174**
Recurrent swelling 91			42 (46)	26	16	371	218 (59)	155	63
Instability despite > 3 months rehab, in									
Sports	**93**		**91 (98)**	**27**	**64**	**376**	**366 (97)**	**107**	**259**
ADL	**92**		**91 (99)**	**6**	**85**	**374**	**366 (98)**	**43**	**323**
Instability (unclear if rehab) in									
Sports	92		40 (43)	22	18	376	165 (44)	118	47
ADL	93		55 (59)	35	20	370	171 (46)	112	59
Patient insists	90		30 (33)	24	6	368	90 (24)	75	15
OS/PT recommends_b_	91		70 (77)	48	22	**371**	**295 (80)**	**194**	**101**
Associated injuries									
Meniscus	**90**		**74 (82)**	**31**	**43**	368	267 (73)	160	107
Articular Cartilage	90		52 (58)	33	19	367	221 (60)	137	84
Ligament	**90**		**74 (82)**	**35**	**39**	363	267 (74)	159	108

Answer options 0 = “no surgery” to 3 = “surgery” for orthopaedic surgeons, and 0 = “low probability” to 3 = “high probability” (to recommend surgery) for physical therapists. Bold-marked factors are considered important with clinical agreement (> 80% of raters answered “2” or “3”). Number of ratings for “2” or “3” are presented. Percentage is calculated for the total amount of respondents for each factor

*ACL-R* anterior cruciate ligament reconstruction, *ADL* activities of daily living, *OS* orthopaedic surgeon, *PT* physical therapist

^a^The total number of respondents for each factor

^b^Orthopaedic surgeons were asked about the factor “responsible physical therapist recommends reconstruction” and vice versa

Fifty-six of the 98 orthopaedic surgeons (57%) answered the open-ended question (Table 3).

**Table 3:** Factors revealed from the open-ended question

Categories Subcategories	Orthopaedic surgeons, total amount of times: 89		Physical therapists, total amount of times:583	
	Single: *n* (%)	Combination: *n*^a^ (%)	Single: *n* (%)	Combination: *n*^b^ (%)
Instability				
Subjective^c^	11 (12)	8 (8)	47 (8)	55 (9)
Rehab^d^	12 (13)	9 (10)	73 (12)	47 (8)
Working^e^	2 (2)	5 (6)	9 (1)	35 (6)
ADL^f^	3 (3)	5 (6)	19 (3)	46 (8)
Patient focus				
Wishes^g^	0	4 (4)	4 (< 1)	20 (3)
QOL^h^	0	0	5 (1)	11 (2)
Symptoms^i^	0	1 (1)	2 (< 1)	20 (3)
Activity demands^j^	6 (7)	12 (13)	55 (9)	114 (22)
Other injuries				
New injuries^k^	0	1 (1)	2 (< 1)	2 (< 1)
Associated injuries^l^	1 (1)	2 (2)	1 (< 1)	2(< 1)
Sociodemographic factors				
Age^m^	0	1 (1)	1(< 1)	15
Gender^n^	0	0	0	1 (< 1)
Objective instability^o^	1 (1)	5 (6)	1 (< 1)	3 (< 1)

Categorization of open-ended question of which factor, or combination of factors that are the most important to recommend ACL-R, including frequency of statements for each category. Each category is divided into “stated as a single factor” and “stated as combination of factors”

*ACL-R* anterior cruciate ligament reconstruction, *ADL* activities of daily living, *QOL* quality of life

^a^*n* = 89. The total amount of times that orthopaedic surgeons mention factors. Percentage is calculated as percentage of the total amount of times as factors was mentioned

^b^*N* = 583. The total amount of times that physical therapists mention factors. Percentage is calculated as percentage of the total amount of times as factors were mentioned

^c^Patient reports instability

^d^Instability despite an adequate rehabilitation period

^e^Instability in working situation

^f^Instability in ADL

^g^Patient’s wishes and/or motivation for ACL-R

^h^Patients experience affected/lowered quality of life

^i^Symptoms as pain and/or swelling

^j^Wishes to return to high knee impact activity/high activity level or risk of involuntarily lowered activity

^j^Risk of new injuries/symptoms (e.g., meniscal tear, developing early osteoarthritis)

^l^Associated injuries on meniscus or ligaments

^m^Young age as a factor for ACL-R

^n^Female sex as a factor for ACL-R

^o^Knee laxity tests measured in clinical setting

Three hundred and thirty-seven of the 391 physical therapists (86%) answered the open-ended question (Table 3). A few comments were more specific to a certain activity, such as recommending ACL-R for patients that compete in alpine skiing or gymnastics, or for young women participating in soccer or floorball. It was reported as “instability in relation to the demands of the knee” or “return to activity with high risks or knee-demanding activity” depending on whether instability was mentioned in combination with the desire to return to the activity (Table 3).

**Fig. 1:**
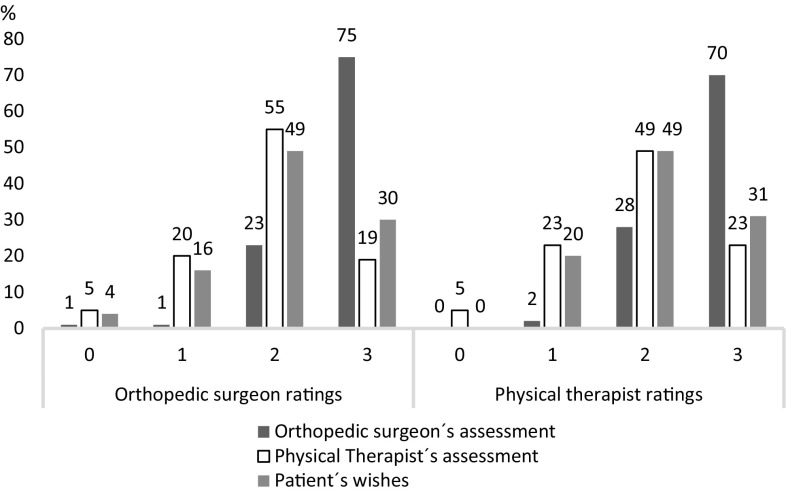
Orthopaedic surgeons and physical therapists ratings of; “To which degree do you believe the decision to treat a patient by ACL-R is based upon the orthopaedic surgeons/physical therapists’ assessments, and the patient’s wishes”. Rating scale 0–3, anchored “to a very low extent” and “to a very high extent”. Figures are expressed as percentage of the total amount of responses

Orthopaedic surgeons and physical therapists had very similar ratings for the impact of their own, each other’s assessments, and the patient’s wishes in the decision-making to recommend ACL-R (Fig. 1).

Regarding compliance to rehabilitation, 73 of 92 (79%) orthopaedic surgeons stated that they often seek information about it, and do so by asking the patient. Sixty-five of 91 (71%) orthopaedic surgeons stated that they sometimes or often read the patient’s medical record to seek information about rehabilitation compliance, and 65 of 92 (70%) stated that they sometimes or often have contact with the treating physical therapist.

Regarding rehabilitation outcome, most of the orthopaedic surgeons (87 of 91, 96%) sometimes or often seek information about the outcome by asking the patient. Sixty-nine surgeons (78% of 89) sometimes or often obtain the information by reading the patient’s medical record, 74 surgeons (81% of 91) sometimes or often contact the treating physical therapist.

Eighty per cent of the physical therapists (296 of 368) stated that when they recommend ACL-R, they often communicate their recommendation to the patient, and 67% (234 of 348) sometimes or often communicate their recommendation to the orthopaedic surgeon.

## Discussion

The most important finding of this study was that Swedish orthopaedic surgeons and physical therapists show agreement that young age, frequent physical activity participation (in work or spare time), and significant knee instability after a period of rehabilitation (> 3 months) are key ACL-R decision-making factors. The results from orthopaedic surgeons are similar to previous studies and existing guidelines [19, 25], although the findings of the present study provide previously unknown information about physical therapists opinions, showing that both professions share a common ground in the values of factors that are important for recommending ACL-R. The factors—open physes, recurrent swelling and instability in sports and ADL when rehabilitation attempts are unclear—received ratings that were the most diverse within professions. This might suggest that these factors in isolation are not considered important in the choice to recommend ACL-R. The factor sex was rated as equally important for both male and female patients, indicating that it does not affect the choice to recommend ACL-R.

The three factors that orthopaedic surgeons and physical therapists agreed were most important to the recommendation for ACL-R were: desire to return to contact or pivoting sports at high/elite level, instability in sports, or instability in ADL despite > 3 months of rehab. This supports previous studies that have identified activity demands and instability as important factors [18–20]. Orthopaedic surgeons considered that a physically demanding occupation was a very important factor for recommending surgery (grading “3” twice as many times compared to “2”). Physical therapists also considered physically demanding occupation important, but not to the same extent (equal amount of “2” and “3” grades) which could be due to the fact that physical therapists in Sweden do not handle sick leave certificates and questions about return to work to the same extent as orthopaedic surgeons do (in Sweden, physical therapists do not write sick leave certificates).

Physical therapists rated patient suffers from significant knee joint instability in sports participation, despite rehabilitation (> 3 months) as very important (grading “3” twice as many times compared to “2”), but among orthopaedic surgeons that factor was less important (equal amount of “2” and “3” grades). That might reflect that physical therapy is often focused on rehabilitation training aimed at return to physical activity and sports participation [30].

Orthopaedic surgeons and physical therapists did not always reach clinical agreement about the importance of factors. Orthopaedic surgeons agreed that associated meniscus or ligament injuries were important factors to recommend ACL-R, while physical therapists did not agree regarding these factors. Physical therapists agreed that orthopaedic surgeon recommended ACL-R is an important factor, while orthopaedic surgeons rated physical therapist recommends ACL-R, slightly lower, (77% agreement).

There was some “clinical disagreement” between orthopaedic surgeons and physical therapists, where about half of the respondents considered the factor as important for recommending ACL-R, while the other half rated it as not important (grades 1 or 2). There was clinical disagreement among orthopaedic surgeons more often than among physical therapists. Open physes (previously rated as a factor that was not important for recommendation of ACL-R according to American orthopaedic surgeons [19]) was in the present study rated as important by 43% of orthopaedic surgeons, and by 20% of physical therapists. The potential for growth abnormality or growth arrest following ACL-R in children with open physes is debated, and consequently, there is strong debate about whether ACL-R is indicated or not [3, 5]. Open physes was among the factors with the highest disagreement among orthopaedic surgeons, which could reflect this uncertainty. It could also reflect that the factor might not be important alone, but could affect the decision if an ACL-R should be recommended due to other factors. Another factor that had high clinical disagreement among orthopaedic surgeons was associated injuries to articular cartilage. Physical therapists disagreed on the same factors as orthopaedic surgeons, except for open physes, where 81% of the physical therapists rated it as not important.

Instability/giving way was consistently reported as the most important factor for recommending ACL-R, which is consistent with previous research [2, 19, 20, 25]. Orthopaedic surgeons and physical therapists often reported instability despite adequate rehabilitation as a single factor influencing their treatment decision-making, which is in line with Swedish recommendations [1]. Orthopaedic surgeons and physical therapists placed slightly different emphasis on the importance of activity demands. Physical therapists rated activity demands as the single most important factor (e.g., patients returning to high knee impact activity) and considered it important as part of a combination of important factors (generally in combination with instability) to a greater extent than orthopaedic surgeons. There is argument that ACL-R increases the chance of patients returning to previous sports activity and level, and lowers the risk of instability and giving way, compared to nonsurgical treatment [5]. However, the literature is not definitive, and other studies have shown that ACL-R might not be necessary to return to previous sports activity and level [10, 11].

It is common for orthopaedic surgeons and physical therapists to collaborate in the treatment of musculoskeletal conditions and for orthopaedic surgeons to refer patients to physical therapists [8]. Orthopaedic surgeons and physical therapists highly rated the impact of the other profession’s assessment in the decision-making. This is important in the light of previous studies that show that collaborative practice have a positive effect on inter-professional communication, and can enhance the quality of care [17].

Orthopaedic surgeons and physical therapists also valued the patient’s wishes in the treatment decision-making process. Shared decision-making in health care is important [6, 12, 24, 28], and a shared decision leads to better treatment results [15]. But even with the patient being involved in the decision process, there is no guarantee that he/she is informed of the options in a satisfying way [29, 30]. Therefore, an evaluation of how patients with ACL injury consider their role in the treatment decision-making process would complete the present study.

Since this study is conducted in Sweden, the generalizability to other contexts might be questionable. In Sweden, it is common for collaboration between orthopaedic surgeons and physical therapists, which improves communication [17], but might not be as common in other countries.

Despite thorough reviewing by an expert panel and pilot testing, the construction of the survey may not have been optimal for the rating of factors influencing the treatment decision. Answer options for the factors in the survey were anchored “no surgery” and “surgery” for orthopaedic surgeons and “little probability that I would recommend ACL reconstruction” and “great probability that I would recommend ACL reconstruction” for physical therapists. The “surgery”/“great probability that I would recommend ACL reconstruction” option was intended to indicate that the factor was important for recommending surgery as treatment, but it might have been possible that the “no surgery” option for orthopaedic surgeons was either interpreted as a factor important to not choose surgery, or that the factor was not important in the recommendation of ACL-R. Because of this, we excluded the “no surgery” data from our analysis to avoid misinterpreted data and improve the reliability of the analyzed data.

Providing predetermined factors may have directed respondents to certain answers. The complexity of a clinical decision is not easily summed up in a survey, and consequently, the result might be a simplified reflection of how the real life decision to recommend ACL-R is taken [19]. To account for this, explore some of the complexity of the decision-making process, we included the open-ended question, which gave a slightly different picture of the level of importance put on different factors.

The intention was to reach as many Swedish orthopaedic surgeons and physical therapists who treated patients with ACL injury as possible. Since almost all orthopaedic surgeons who perform ACL-R in Sweden, are registered in the Swedish National Knee Ligament Register, we believe we have reached the majority of the orthopaedic surgeons that perform ACL-R in Sweden. There are approximately 21,000 licenced physical therapists in Sweden working across different settings [26]. There is no way of knowing to what extent we succeeded in reaching physical therapists treating patients with ACL injury, since there is no specific registration of these physical therapists. Because of that, an accurate response rate is uncertain.

This study shows that orthopaedic surgeons and physical therapist mainly agree on factors that are important to recommend ACL reconstruction, with some slight differences which might be due to different point of views. Physical therapists often meet each patient more frequently, and might, therefore, emphasise other aspects than the orthopaedic surgeons, in the treatment choice. Collaboration between the two groups could be valuable in the choice of which treatment to recommend, to enlighten different aspects and thus provide the best recommendations for the patients.

## Conclusion

Orthopaedic surgeons and physical therapists rated patients’ wishes to return to contact/pivoting sports, knee joint instability in physical activity or activities of daily living despite adequate rehabilitation, physically demanding occupation, and young age as important factors for their decision to recommend ACL-R. The most prominent single factor influencing the decision was “knee instability”. Both professions consider their own and each other’s assessment as important for the decision to recommend ACL-R.

## Electronic supplementary material

Below is the link to the electronic supplementary material.

Supplementary material 1 (DOCX 26 KB)

Supplementary material 2 (DOCX 22 KB)

## Abbreviations

ACL: Anterior cruciate ligament
ACL-R: Anterior cruciate ligament reconstruction
