# Supplementary material for: Activity demands and instability are the most important factors for recommending to treat ACL injuries with reconstruction
Source: Knee Surg Sports Traumatol Arthrosc. 2018 Feb 6;26(8):2401–9. doi: 10.1007/s00167-018-4846-1 (PMC6061764; doi:10.1007/s00167-018-4846-1)
Supplement: Supplementary file 2 — Supplementary material 2 (DOCX 22 KB) [file 167_2018_4846_MOESM2_ESM.docx]

I have read the written information about the study:

- I have received written information about the aim of the study and consent to participate
- I do not treat this group of patients and therefore decline participation
- I decline participation

Age

- <30
- 31-45
- 46-55
- >55

Gender

- Male
- Female

Do you have extended education in ACL injury rehabilitation? (E.g. shorter courses)

- Yes
- No

For how long have you been working with ACL-deficient patients (non-reconstructed)?

- <2 years
- 2-5 years
- 6-10 years
- > 10 years

How many ACL-deficient or ACL reconstructed patients have you rehabilitated during the last 6 months? (Both ongoing and discharged patients)

- 0
- 1-4
- 5-15
- >15

Do you have knowledge about guidelines/health care programs outlining which patients should undergo an ACL reconstruction? (Multiple answers are allowed)

- No, I have no knowledge of such guidelines or health care programs
- Yes, there are local guidelines/health care programs
- Yes, there are national guidelines
- Yes, there are international guidelines

How do the following factors affect your assessment of whether a patient should undergo ACL reconstruction (0= little probability that I would recommend ACL reconstruction, 3= great probability that I would recommend ACL reconstruction)

| Patient’s age | |  |  |  |  |
| --- | --- | --- | --- | --- | --- |
|  | <25 years | 0 | 1 | 2 | 3 |
|  | 25-40 years | 0 | 1 | 2 | 3 |
|  | >40 years | 0 | 1 | 2 | 3 |
| Patient has open physes | | 0 | 1 | 2 | 3 |
| Patient’s sex | |  |  |  |  |
|  | Male | 0 | 1 | 2 | 3 |
|  | Female | 0 | 1 | 2 | 3 |
| Patient wishes to return to: | |  |  |  |  |
|  | Contact sports/pivoting sports at high/elite level | 0 | 1 | 2 | 3 |
|  | Contact sports/pivoting sports at recreational level | 0 | 1 | 2 | 3 |
|  | Non-contact sport at high/elite level | 0 | 1 | 2 | 3 |
|  | Non-contact sport at recreational level | 0 | 1 | 2 | 3 |
| Patient’s occupation is physically demanding for the knee and requires knee stability | | 0 | 1 | 2 | 3 |
| Patient suffers from recurrent swelling of the knee joint | | 0 | 1 | 2 | 3 |
| Patient suffers from significant knee joint instability in sports participation, despite rehabilitation (>3 months): | |  |  |  |  |
|  | Significant instability in sports participation | 0 | 1 | 2 | 3 |
|  | Significant instability in activities of daily living | 0 | 1 | 2 | 3 |
| Patient suffers from significant knee joint instability in sports participation, unclear if rehabilitation (>3 months) is completed: | |  |  |  |  |
|  | Significant instability in sports participation | 0 | 1 | 2 | 3 |
|  | Significant instability in activities of daily living | 0 | 1 | 2 | 3 |
| Patient, or relative, insists on surgery | | 0 | 1 | 2 | 3 |
| Responsible orthopedic surgeon recommends surgery  Significant associated injuries | |  |  |  |  |
|  | Meniscus | 0 | 1 | 2 | 3 |
|  | Articular cartilage | 0 | 1 | 2 | 3 |
|  | Ligament injuries | 0 | 1 | 2 | 3 |

Please write in your own words the single most important factor, or combination of factors, that you consider when recommending a patient to undergo an ACL reconstruction:

______________________________________________________________

If you recommend ACL reconstruction, how do you present your assessment?

- By discussing/informing the patient
- By discussing/informing the orthopedic surgeon
- Others:_________________________

To which degree do you believe that the decision to do ACL reconstruction is based upon: (0= to a very low degree, 3= to a high degree):

| The physical therapist’s assessment? | 0 | 1 | 2 | 3 |
| --- | --- | --- | --- | --- |
| The orthopedic surgeon’s assessment? | 0 | 1 | 2 | 3 |
| The patient’s wishes? | 0 | 1 | 2 | 3 |
